# Sir John Floyer and the Physician's Pulse-Watch

**Published:** 1906-02-24

**Authors:** 


### Sir John Floyer and the Physician's Pulse-Watch.

The art of feeling the pulse only dates in modern times from the year 1707, when Sir John Floyer published "The Physician's Pulse-watch; or, an essay to explain the old art of feeling the pulse and to improve it by the help of a pulse watch." At first sight this appears surprising, but a little reflection will show that a modern record of the pulse deals rather with its rapidity than its quality, and to measure rapidity the sand-glass and water-clocks of our forefathers were wholly inadequate. Minute hands were not generally used on watches until 1665, and second hands only came into favour in 1761. Hippocrates was not ignorant of the nature of the pulse, but Galen was the great inventor of the art of studying the pulse, although he erred in considering it rather from a philanthropic than from a clinical standpoint. The Galenic school and its classification of the pulse held sway in medicine for many hundred years until its authority was swept away by the discoveries of Harvey and the Oxford school of anatomists in the seventeenth century. Sir John Floyer, as the exponent of this school, treated of the pulse clinically and numerically for he says: "All I pretend to is the discovery of a rule whereby we may know the natural pulse, and the excesses and defects from that in diseases; and from the pulse we may take our indications for the use of Diet and medicines." Sir John Floyer conducted his experiments on the pulse along purely modern lines of research. He first imitated the pulse and circulation by an artificial schema, for "I injected into the small guts of a cow a sufficient quantity of water to fill them: and I laid the guts round on the grass in three or four rings: the one end of the gut was fastened to an engine [pump] which was set in a pail of water . . . I observed that the circle which was next the pump vibrated as oft as the water was injected, and that the water moved forward upon every stroke of the pump and returned back a little after the force was spent. And this regurgitation may be perceived in the pulse of weak persons and in obstructions of the artery. . . . I observed further that the water was forced out of the guts in a continual stream, and every stroke of the pump did accelerate and give a jerk to the liquor like the bleeding from an artery cut. The gut was always full of water, and when I laid a brick upon the circle of it the pulse in the gut felt hard. The force of the water injected protruded the gut and the annular fibres by their material restitution promoted the motion of the water and kept the stream from any interruption, though the injection was made by intervals." From this clear demonstration of the physiological cause of the pulse Sir John Floyer proceeded to investigate the rapidity of the pulse in healthy bodies at different ages and under varying conditions, personal and atmospheric. He says of himself: "I will give the following observations I made on my own pulse in September last. The morning pulse fasting was 76, after rubbing with brushes 86, and this did stand so some time, but at last returned to the morning pulse, which fell to a lower number before dinner. After dinner the pulses were 89, and before supper 83. Another day the pulse before dinner was 77, and after dinner it was 95. I drank some ale after dinner and two dishes of coffee after it: by both the pulse was raised more than ordinarily it is after eating. The night before an asthma fit I generally observe my pulse about 90, and in the fit at least 95 . . . I give this instance to show how we may know our diseases approach by the number of the pulses, and by the same we may discern the degrees by which it goes off. . . . The slower my pulse beats, the better is my breath, but if my pulse be 90, I am always passive, but 95 makes me asthmatic; I am of lean habit." On March 26, 1706, Sir John counted the pulses at the following ages: "Three years old a fair boy with a moderate habit 93 pulses in a morning in one minute; five years old in a girl of a thin habit and brown hair 106; six years old a girl subject to a cough 105." On the same day he counted the pulses of 21 other children of varying ages, and to one child he gave a glass of sack which raised his pulse to 90 beats instead of the normal 69-76. Many of his experiments were carried out in the hospital at Lichfield. Sir John concludes his essay with an account of the Chinese art of feeling the pulse, to which is added an extract out of Andrew Cleyer on the same subject.

Floyer was born at Hintes in Staffordshire in April 1649, the second son of Richard Floyer, and was educated at Queen's College, Oxford, where he graduated B.A. in 1668, and M.D. in 1680. He practised at Lichfield, and married Mary Fleetwood, a widow. He was knighted 1685, and died February 1, 1734. It was Sir John Floyer who sent Dr. Samuel Johnson to London that he might be touched by Queen Anne for the cure of his scrofula, or king's evil; it was of Sir John Floyer that the great lexicographer wrote to Mr. Langton March 27, 1784, complaining of his asthma: "Sir John Floyer whom the physical race consider as an author of one of the best books upon the affection panted on to 90 as was supposed; and why were we content with supposing a fact so interesting of a man so conspicuous? Because he corrupted at perhaps 70 or 80 the register that he might pass for a younger man than he was. He was not much less than 80 when to a man of rank who modestly asked his age he answered: 'Go look,' though he was in general a man of civility and elegance." Dr. Johnson's accusation, however, is quite groundless. The register of his birth has not been altered and the transcripts from the registers of the University of Oxford show that he was really born in 1649. He was 85, therefore, when he died. It is noteworthy, too, that Dr. Johnson's father, Michael Johnson, published Dr. Floyer's first book whose title page reads "The Touchstone of Medicines, printed for Michael Johnson, bookseller, and are to be sold at his shops at Lichfield and Uttoxeter in Staffordshire, and at Ashby-de-la-Zouch in Leicestershire, 1687."

